# Poster Session II - A257 THE ROLE OF BACTERIAL LPC AND LPA IN CHRONIC ABDOMINAL PAIN: A LONGITUDINAL STUDY IN PATIENTS WITH IBD

**DOI:** 10.1093/jcag/gwaf042.257

**Published:** 2026-02-13

**Authors:** J S Alawfi, J Pujo, F A Vicentini, G Rueda, M Quaderi, V Mohan, M Hall-Bruce, A Nardelli, G De Palma, S Collins, P Bercik

**Affiliations:** Gastroenterology, Farncombe Family Digestive Health Research Institute, Hamilton, ON, Canada; Gastroenterology, Farncombe Family Digestive Health Research Institute, Hamilton, ON, Canada; Gastroenterology, Farncombe Family Digestive Health Research Institute, Hamilton, ON, Canada; Gastroenterology, Farncombe Family Digestive Health Research Institute, Hamilton, ON, Canada; Gastroenterology, Farncombe Family Digestive Health Research Institute, Hamilton, ON, Canada; Gastroenterology, Farncombe Family Digestive Health Research Institute, Hamilton, ON, Canada; Gastroenterology, Farncombe Family Digestive Health Research Institute, Hamilton, ON, Canada; Gastroenterology, Farncombe Family Digestive Health Research Institute, Hamilton, ON, Canada; Gastroenterology, Farncombe Family Digestive Health Research Institute, Hamilton, ON, Canada; Gastroenterology, Farncombe Family Digestive Health Research Institute, Hamilton, ON, Canada; Gastroenterology, Farncombe Family Digestive Health Research Institute, Hamilton, ON, Canada

## Abstract

**Background:**

Patients with inflammatory bowel disease (IBD) often experience abdominal pain, despite being in remission, and many associate their pain with the intake of certain foods. Emerging evidence links lysophosphatidylcholine (LPC) and lysophosphatidic acid (LPA) to neuropathic pain, with preclinical studies suggesting that gut bacteria are able to produce these compounds from dietary phosphatidylcholine (PC).

**Aims:**

To study the association between fecal LPC and LPA, abdominal pain, and dietary PC intake in patients with IBD.

**Methods:**

IBD patients in remission (determined endoscopically and/or by fecal calprotectin<250 mg/kg) with chronic abdominal pain were enrolled in a longitudinal study. Pain was assessed by the Visual Analog Scale (VAS), and stool samples were collected daily for at least one week; diet was studied by a 3-day diary. Fecal LPC and LPA were extracted using Bligh-Dyer and solid phase extraction techniques and analysed using Liquid Chromatography-Mass Spectrometry (LC-MS). Dietary intake, focused on total choline and PC, was assessed by ESHA software.

**Results:**

Eight patients (female=6; Crohn’s disease=2, UC = 6; age 43.5 [endif]-->5.3 years) completed the study. Patients with previous bowel surgery reported higher pain scores compared to those without surgery (49.8 [endif]-->8.9 vs 19.7 [endif]-->9.2). Fecal LPA and LPC were elevated in periods of high pain compared to low pain (Table 1). The estimated choline intake was 358.8 [endif]-->48.4 and 297.8 [endif]-->29.3 mg/d in males and females, respectively (adequate intake 550 and 425 mg/d). The estimated PC intake was 233.0 [endif]-->43.8 and 160.1 [endif]-->24.3 mg/d in males and females, respectively. There was no clear association between intake of PC and pain scores.

**Conclusions:**

IBD patients with colitis in remission suffer from chronic abdominal pain, more severe in those with previous bowel resection. Fecal LPC and LPA concentrations fluctuate, with higher levels associated with high pain. Patients with IBD have low choline intake, possibly unconsciously avoiding dietary substrates for bacterial LPC and LPA production.

**Funding Agencies:**

Crohn’s Colitis Foundation (US), J.A received Ph.D scholarship from Imam Abdulrahman Bin Faisal University, Dammam, Saudi Arabia.

**A257 Table.1:** Fecal LPA and LPC concentrations ug/g stool

	Total LPC^**^	LPC 16.0^**^	LPC 18.0^**^	Total LPA^*^	LPA 18.0^*^	LPA 20.4^*^
High pain	30.1 ±6.2	15.5 ±3.1	7.6±1.5	22.9 ±3.6	9.5 ±1.7	0.13 ±0.03
Low pain	3.1 ±0.9	1.5 ±0.6	0.9±0.2	13.3 ±2.3	4.3 ±0.9	0.35 ±0.09

Data presented as mean ±SEM,

* p < 0.05,

** p < 0.01

