# Correction to: Poster Session II - A258 THE ROLE OF BACTERIAL LPC AND LPA IN CHRONIC ABDOMINAL PAIN: A LONGITUDINAL STUDY IN PATIENTS WITH IBD

**DOI:** 10.1093/jcag/gwag012

**Published:** 2026-04-16

This is a correction to: J S Alawfi, J Pujo, F A Vicentini, G Rueda, M Quaderi, V Mohan, M Hall-Bruce, A Nardelli, G De Palma, S Collins, P Bercik, Poster Session II - A258 THE ROLE OF BACTERIAL LPC AND LPA IN CHRONIC ABDOMINAL PAIN: A LONGITUDINAL STUDY IN PATIENTS WITH IBD, *Journal of the Canadian Association of Gastroenterology*, Volume 9, Issue Supplement_1, February 2026, gwaf042.257, https://doi.org/10.1093/jcag/gwaf042.257

In the originally published version of this abstract, presenter J.S. Alawfi’s affiliation with the “Department of Clinical Nutrition, College of Applied Medical Sciences, Imam Abdulrahman Bin Faisal University, Dammam, Saudi Arabia, Dammam, Saudi Arabia” was inadvertently omitted.

This error has been corrected, and the published abstract has been updated to show the presenter’s affiliations as follows:

J.S. Alawfi^1, 2^

^1^Gastroenterology, Farncombe Family Digestive Health Research Institute, Hamilton, ON, Canada; ^2^Department of Clinical Nutrition, College of Applied Medical Sciences, Imam Abdulrahman Bin Faisal University, Dammam, Saudi Arabia, Dammam, Saudi Arabia.

